# Influence of Silver as a Catalyst on the Growth of β-Ga_2_O_3_ Nanowires on GaAs

**DOI:** 10.3390/ma13235377

**Published:** 2020-11-26

**Authors:** Badriyah Alhalaili, Howard Mao, Daniel M. Dryden, Hilal Cansizoglu, Ryan James Bunk, Ruxandra Vidu, Jerry Woodall, M. Saif Islam

**Affiliations:** 1 Nanotechnology and Advanced Materials Program, Kuwait Institute for Scientific Research, 13109 Kuwait City, Kuwait; homa@ucdavis.edu (H.M.); hcansizoglu@ucdavis.edu (H.C.); 2 Electrical and Computer Engineering, University of California at Davis, Davis, CA 95616, USA; dmdryden@ucdavis.edu (D.M.D.); rjbunk@ucdavis.edu (R.J.B.); jwoodall@ucdavis.edu (J.W.); sislam@ucdavis.edu (M.S.I.); 3 Materials Science and Engineering, University of California at Davis, Davis, CA 95616, USA; 4 Faculty of Materials Science and Engineering, University POLITEHNICA of Bucharest, 060042 Bucharest, Romania

**Keywords:** Ga_2_O_3_, GaAs, nanowires, oxidation, silver catalyst

## Abstract

A simple and inexpensive thermal oxidation process was performed to synthesize gallium oxide (Ga_2_O_3_) nanowires using Ag thin film as a catalyst at 800 °C and 1000 °C to understand the effect of the silver catalyst on the nanowire growth. The effect of doping and orientation of the substrates on the growth of Ga_2_O_3_ nanowires on single-crystal gallium arsenide (GaAs) wafers in atmosphere were investigated. A comprehensive study of the oxide film and nanowire growth was performed using various characterization techniques including XRD, SEM, EDS, focused ion beam (FIB), XPS and STEM. Based on the characterization results, we believe that Ag thin film produces Ag nanoparticles at high temperatures and enhances the reaction between oxygen and gallium, contributing to denser and longer Ga_2_O_3_ nanowires compared to those grown without silver catalyst. This process can be optimized for large-scale production of high-quality, dense, and long nanowires.

## 1. Introduction

Ga_2_O_3_ has attracted the attention of scientists due to its unique properties including a wide bandgap of 4.9 eV, a high melting point of 1900 °C, excellent electrical conductivity, and both high thermal and chemical stability [1,2]. These features have led to the consideration of Ga_2_O_3_ nanowires as a useful material for applications in power electronics, solar-blind UV detectors, and device applications in harsh environments [3,4].

Ga_2_O_3_-based materials are attractive in semiconductor industry. However, to reduce the cost of Ga_2_O_3_ wafers is a major challenge. Many techniques have been performed to synthesize thin film and nanowires of Ga_2_O_3_, including thermal oxidation [5,6], plasma-assisted molecular beam epitaxy (MBE) [7,8,9], pulsed laser ablation [10,11], chemical vapor deposition (CVD) [12,13] and the hydrothermal synthesis [14,15,16].

Thermal oxidation of GaAs is a simple and inexpensive method to grow Ga_2_O_3_ nanowires that has been examined by many research groups [6,17,18,19,20]. At temperatures beyond 600 °C [6,20], the components of GaAs decompose, leading to phase separation and formation of β-Ga_2_O_3_. In sequence, as the Arsenic (As) detaches from the surface of GaAs, it evaporates, and Ga melts down to produce hemispherical droplets of liquid Ga at the surface of GaAs [20]. The residual oxygen in the chamber interacts with the liquid Ga droplets to develop thin film of Ga_2_O_3_ particularly at temperatures in the range of 585–750 °C. When temperatures increase above 750 °C, Ga_2_O_3_ nanowire growth starts to dominate on the surface of Ga_2_O_3_ [6]. When the temperature is increased further, the length, height, and density of nanowires increase.

However, the use of Ag catalyst adds a new dimension to the growth kinetics of Ga_2_O_3_ nanowires due to the simultaneous interactions of oxygen and silver with GaAs at the surface. The Ga_2_O_3_ nucleation and growth of nanowires by using Ag catalyst could be influenced by temperature. In order to assess the effect of Ag catalyst, Ag on GaAs was oxidized at different temperatures both under and above 800 °C to measure the effect of Ag’s melting temperature (961.8 °C) on the growth of Ga_2_O_3_ nanowires.

Although silver nanoparticles have been utilized in the past as a possible catalyst for Ga_2_O_3_ nanowire growth [21,22,23,24], the use of thin films of Ag as a catalyst for Ga_2_O_3_ nanowire growth on GaAs substrate has not been investigated. Ag thin films could be a potential alternative to nanoparticles, as a catalyst for Ga_2_O_3_ nanowire growth.

In this work, we propose a simple and inexpensive thermal oxidation process to produce Ga_2_O_3_ nanowires using Ag thin film as a catalyst. The thin film annealing method was used to generate metal nanoparticles as a catalyst for nanowire growth. Using this method, first a silver thin film is deposited onto a substrate. When the substrate is heated, the thin film dewets form nanoparticles. The benefit of first using a thin film [25] instead of directly applying nanoparticles is that it maximizes the size and density of the nanoparticles. Consequently, we observed the enhancement of the Ga_2_O_3_ nanowire growth by Ag films from the thermal oxidation of a GaAs substrate. Hence, we analyze the properties and characteristics of the Ga_2_O_3_ nanowires obtained using Ag as a catalyst on GaAs substrate.

## 2. Materials and Methods

First, 1 cm^2^ GaAs samples were cleaned with acetone and ethyl alcohol (University Wafers, Boston, MA, USA), rinsed with deionized water, and dried with an N_2_ gun. GaAs substrates used in this experimental work were as follows: n-type (Si-doped mono-crystalline GaAs labeled N111) and p-type (Zn-doped monocrystalline GaAs labeled P111). Then, 50 nm silver thin films were deposited on the samples using a Lesker sputtering system (Kurt J. Lesker Company, Jefferson Hills, PA, USA). Both Ag-coated and Ag-free GaAs samples were used to grow nanowires under the same conditions, where each sample was oxidized separately one after the other to avoid contaminations.

**Figure 1:**
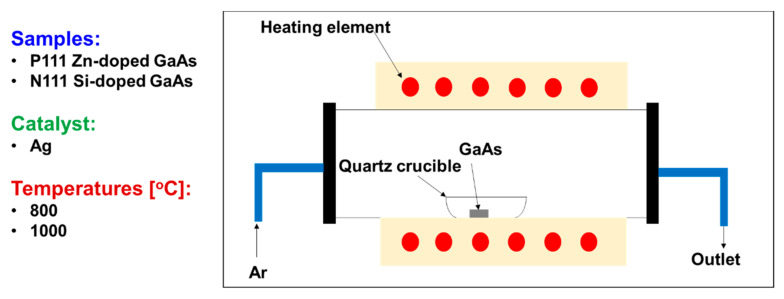
Experimental setup for thermal oxidation of GaAs.

Afterwards, the samples were re-cleaned with acetone and ethyl alcohol to remove contaminants, rinsed with deionized water, and dried with an N_2_ gun. Then, they were loaded into an Aluminum oxide crucible which was placed into a horizontal quartz furnace made by MTI Corporation, Richmond, CA, USA. The samples were exposed to temperatures of 800 °C and 1000 °C for 40 min. To set up the system, the temperature for the oxidation experiments was initially defined manually in the oven, depending on the position of the sample inside the tube. The temperature was measured with a thermocouple (MTI Corporation, Richmond, CA, USA). The oxidation process had the following schedule: 40 min for heating the sample to oxidation temperature (i.e., 800 or 1000 °C), 40 min for maintaining the temperature and 1 h for cooling the samples to room temperature. Heating occurred under a 100 sccm argon flow. Figure 1 shows a sample positioned inside the quartz tube while argon gas flows.

Upon cooling of the furnace to room temperature, the samples were removed from the furnace, cleaved and characterized. Scanning electron microscopy (SEM) was used to characterize the morphology of the nanowires. A focused ion beam (FIB) equipped with X-MaxN 50 mm^2^ energy dispersive spectroscopy (FIB/EDS) from Oxford Instruments (Oxford Instruments NanoAnalysis, Concord, MA, USA) was used to perform elemental and chemical microanalysis. A 200 kV JEOL JEM-2500SE high resolution transmission electron microscope (HRTEM) (JEOL USA, Inc., Peabody, MA, USA) and a 200 KeV JEOL JEM-2100AC scanning transmission electron microscope (STEM) (JEOL USA, Inc., Peabody, MA, USA) equipped with EDS were used to focus on the surface of a single nanowire to obtain higher detection capabilities of the decorated nanowire. For the electrical measurements, the electrical contacts were patterned on the top surface of the nanowires using a shadow mask, and then 5 nm Cr and 50 nm Au were sputtered using a Lesker sputtering system. A custom probe station attached to a Keithley 2400 SMU (Tektronix Inc., Beaverton, OR, USA) was used and UV illumination was achieved using a Dymax Bluewave75 UV lamp (280–450 nm) (Dymax Corporation, Torrington, CT, USA) with a light intensity of 15 W/cm^2^.

## 3. Results

### 3.1. Temperature Dependence of the Growth of Ga_2_O_3_ Nanowires

#### 3.1.1. Ga_2_O_3_ Nanowires Morphologies

**Figure 2:**
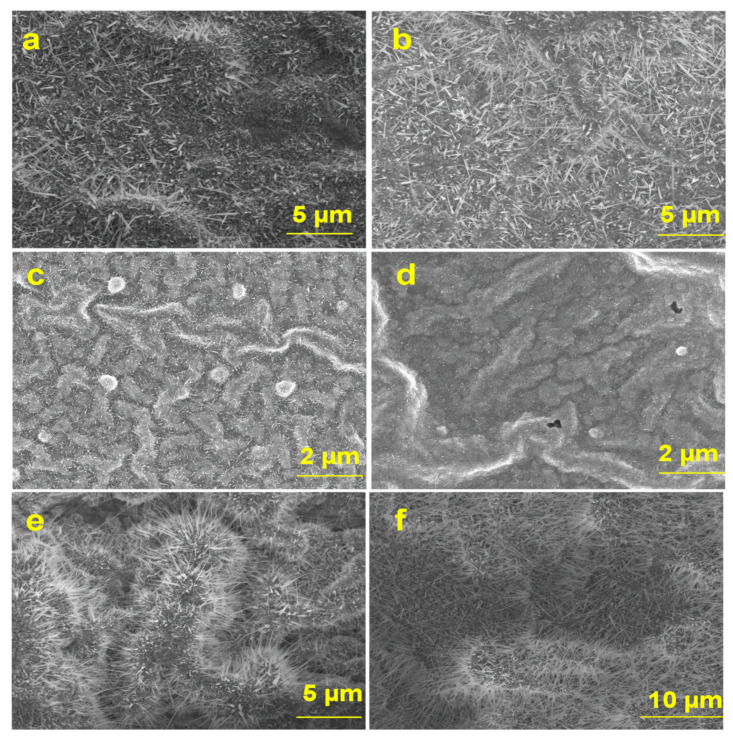
SEM images of Ga_2_O_3_ NWs on Si-doped monocrystalline (N111) GaAs (left column) and Zn-doped monocrystalline (P111) GaAs (right column). Thicker and shorter Ga_2_O_3_ NWs without Ag at 1000 °C on (**a**) (N111) and (**b**) (P111) GaAs substrate. An early stage of Ga_2_O_3_ NWs nucleation at 800 °C on (**c**) (N111) and (**d**) (P111) GaAs substrate coated with Ag. Thinner, longer and denser NWs at 1000 °C on (**e**) (N111) and (**f**) (P111) GaAs substrate coated with Ag. The growth of Ga_2_O_3_ NWs is strongly influenced by Ag catalyst and its melting temperature.

Thermal oxidation was performed at 800 °C and 1000 °C on GaAs substrates that were either coated or not coated with Ag. Representative SEM micrographs of P(111) and N(111) samples without and with Ag thin film coating are presented in Figure 2. The SEM images were acquired at an accelerating voltage of 10 KV and a working distance of 4.8 nm. The morphology of Ga_2_O_3_ nanowires (NWs) on N(111) and P(111) GaAs took place at the early stage of nanowire nucleation at 800 °C, where a small amount of short nanowires were observed. As can be seen in the images, the morphology is strongly influenced by the oxidation temperature.

As the oxidation temperature increases, the density of nanowires also increases. At 1000 °C, the nanowires grown in the presence of Ag on GaAs surface showed different morphology, appearing significantly thinner, longer and more densely packed than on Ag-free GaAs. This observation confirms the effect of Ag on the growth of β-Ga_2_O_3_ nanowires. The Ag catalyst leads to thinner nanowires with diameters between 25 and 40 nm and lengths in several tens of µm. In contrast, the sample without an Ag coating had thicker and shorter nanowires with diameters ranging from 250 to 400 nm and lengths in the range of 800 nm–1.4 µm. As the size of the metal catalyst reduced, the nanowire becomes thinner by the diameter of the catalyst [26,27]. Hence, deposition of Ag thin film enhances denser, thinner and longer Ga_2_O_3_ nanowires on the surfaces during the oxidation of GaAs.

Compared to previous catalysts used in previous studies for the growth of β-Ga_2_O_3_ (Table 1), we obtained longer and thinner nanowires than those previously reported. Additionally, using silver as a catalyst, we obtained nanowires that were thinner than most of the nanowires grown using other catalysts as seen in Table 1. Looking at the diameters of the nanowires, only Ag nanoparticles produced nanowires that were thinner than those grown with an Ag thin film as a catalyst. In terms of length, the nanowires grown using a thin film of Ag were comparable to most of the lengths reported using other catalysts other than Ni and Ag nanoparticles. Table 1 also shows that the effect of Ag catalyst in particular has not been addressed. Thus, using Ag as a catalyst for Ga_2_O_3_ nanowire growth, the thinnest and sharpest nanowires can be obtained.

#### 3.1.2. Ga_2_O_3_ Elemental Composition by EDS

Energy dispersive spectroscopy (EDS) was used to perform elemental and chemical microanalysis on the samples. EDS profile analysis was also performed for different samples with and without of Ag and heated at 800 °C and 1000 °C. Table 2 shows the atomic percentages (at. %) of the elemental Ga, As, O and Ag composition of the samples at 800 °C and 1000 °C, respectively. It is obvious that in both cases the oxygen concentration increases with oxidation temperature; however, the oxygen concentration at 1000 °C was much higher than the oxygen concentration at 800 °C. Additionally, Ag was not detected at 1000 °C and there was much more As at 800 °C than there was at 1000 °C. However, the EDS mapping confirmed the existence of As at 800 °C and the disappearance of As at 1000 °C.

**Figure 3:**
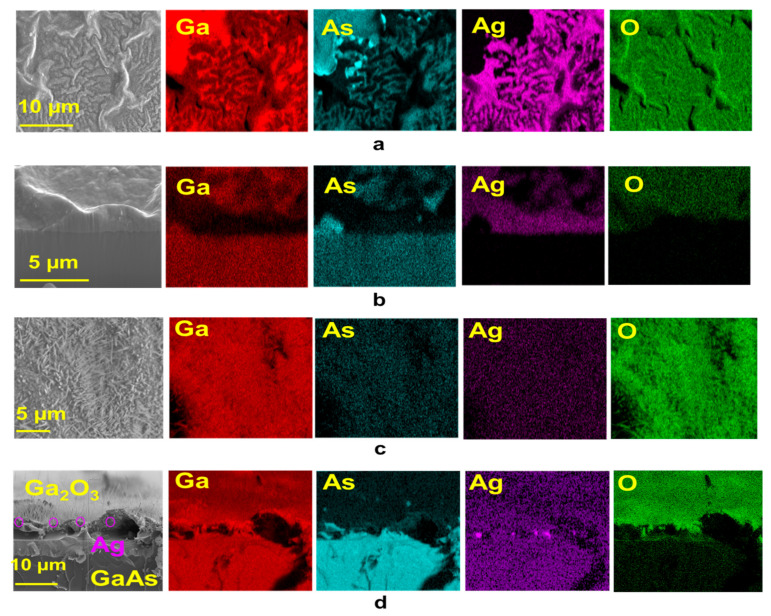
EDS mapping of Ga, As, Ag and O of Ga_2_O_3_ NWs on P(111) Zn-doped GaAs substrate coated with Ag. (**a**) Top views and (**b**) side views of Ga_2_O_3_ NWs grown at 800 °C on GaAs substrate. Ag thin film was dewetted at the surface of GaAs. (**c**) Top views and (**d**) side views of Ga_2_O_3_ NWs grown at 1000 °C on GaAs substrate. Ag thin film was blocked by the coating layer of Ga_2_O_3_ NWs.

Focused ion beam (FIB) equipped with X-MaxN 50 mm^2^ energy dispersive spectroscopy (EDS) from Oxford Instruments was used to perform elemental and chemical microanalysis on the samples. EDS mapping results of oxidized P(111) Zn-doped GaAs coated with Ag at 800 °C and 1000 °C (Figure 3) show the existence of Ag. Interestingly, at 800 °C, the top and side views of Figure 3a show dewetting islands of Ag thin film at the surface of GaAs. In contrast, at 1000 °C, top view images did not show the presence of silver. The surface was completely coated with Ga_2_O_3_ nanowires. Hence, a cleaved cross-sectional analysis of a sample was performed (Figure 3b).

It has been observed that some silver existed at the surface and was buried underneath the film of Ga_2_O_3_ nanowires. In addition, the literature shows that there might be As present in the interface region during the formation of Ga_2_O_3_ [31,32]. According to Ag-GaAs phase diagram, when the temperature is below the melting point of Ag (~962 °C), there will a particular mass of GaAs in Ag; however, at 1000 °C, a mixture of Ag–Ga–As–O in the liquid phase could be formed and lead to supersaturation of the growth process. There is a lack of experimental results that discuss the chemical and physical interactions of the Ag–Ga–As–O system. Based on the Ag–GaAs phase diagram ((No. 1300896) [33]), during the cooling process, GaAs and Ag are the only products that are involved during the cooling path. However, we expect the products of Ag–GaAs under thermal oxidation to be Ga_2_O_3_, Ag, As_(g)_ and GaAs.

#### 3.1.3. XRD Characterization

**Figure 4:**
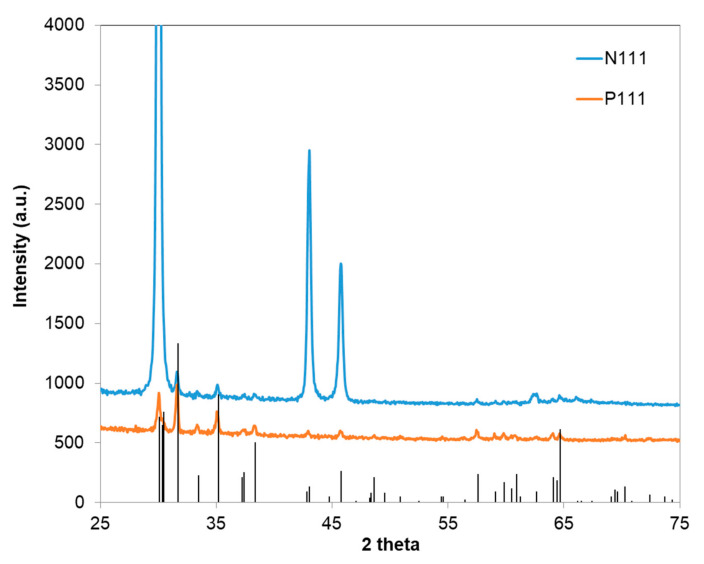
X-ray diffraction spectra of Ag-coated p-type Zn-doped monocrystalline GaAs and n-type Si-doped mono-crystalline GaAs, which were indexed in comparison to PDF 00-041-1103, for β-Ga_2_O_3_ grown at 1000 °C.

XRD was performed with a Panalytical XPert PRO Diffractometer (Malvern Panalytical, Almelo, The Netherlands). Figure 4 shows the XRD patterns of P(111) and N(111) GaAs samples oxidized at 1000 °C. The XRD pattern of both oxidized GaAs samples shows well defined diffraction peaks (400), (110), ($\bar{4}10$), ($\bar{2}02$), (111), ($\bar{1}12$), (003), which correspond to the reflection plane of the monoclinic β-Ga_2_O_3_ (PDF 00-014-4103).

The XRD pattern shows the most intense β-Ga_2_O_3_ peak for the (400) plane, which indicates a preferential a-axis orientation of crystallites. A similar preferential orientation was observed when GaAs was thermally oxidized at 900 °C to obtain β-Ga_2_O_3_ [34]. A preferred [400] orientation was also observed after annealing Ga_2_O_3_ thin film at 1050 °C for 1 h [35]. Annealing the gallium oxide resulted in increased grain size and improved crystallinity, with the (400) peak being the most important of all peaks in the XRD pattern of β-Ga_2_O_3_.

The XRD patterns of the N111 and P111 samples were compared with the standard diffraction pattern of β-Ga_2_O_3_. Comparing the ratio of the intensity of two diffraction peaks (400) and (111) (i.e., I_400_/I_111_) of the β-Ga_2_O_3_ standard bulk with that of the nanowires, it was found that this ratio increased from 0.468 to 6.42 for P111 and to 417.38 for N111. This observation demonstrates that the [400] orientation becomes dominant during thermal oxidation and suggests that the nanowires might have preferential growth in the [400] direction. The growth of nanowires in the direction of the a-axis, which is also the largest lattice parameter of the β-Ga_2_O_3_ monoclinic cell, is accompanied by lateral compression since layered semiconductors are characterized by highly anisotropic elastic properties due to the presence of two types of bonding in the crystal (strong bonding in a layer and weak interlayer bonding) [36].

#### 3.1.4. XPS Characterization

**Figure 5:**
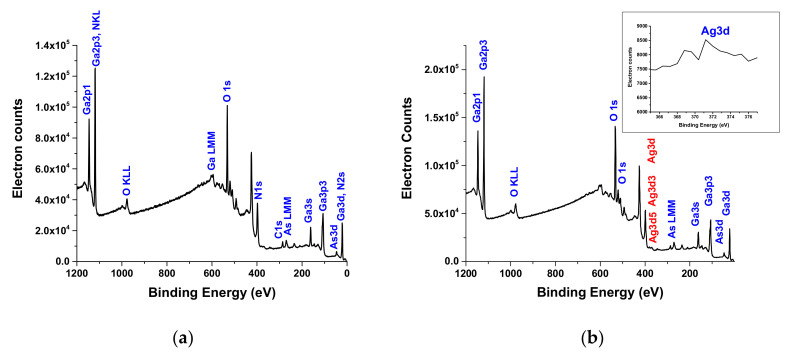
XPS of the β-Ga_2_O_3_ obtained from Zn-doped P(111) GaAs oxidized at 1000 °C: (**a**) no Ag (**b**) coated with 50 nm Ag. The peaks of Ga and Ag have positive slight shifts in energy due to the difference in electronegativity and work function. In addition, as the diameter of Ag decreases, the binding energy increases. Ag has been detected by XPS and the inlet shows the peaks for Ag in the range of metallic Ag from 365 to 377 eV.

X-ray photoelectron spectroscopy (XPS) was performed on a PHI 5800 XPS system to analyze the elemental composition of the surface. Figure 5 shows XPS spectra of the film of Ga_2_O_3_ nanowires obtained by oxidation from GaAs substrate without and with Ag in the binding energy range of 0 to 1200 eV.

The XPS spectrum shows that the chemical composition of the particles at the surface of Ag-free β-Ga_2_O_3_ includes O, Ga and As at the surface of Ga_2_O_3_ in the presence of Ag, which includes O, Ga, As, and Ag. The C peaks originate mostly from atmospheric contamination since the sample was exposed to air. In the case of Ag-free β-Ga_2_O_3_ (Figure 5a), the binding energies of Ga 3d, O 1s and As 3d are 20.6 eV, 532 eV and 47 eV, respectively. However, in the case of β-Ga_2_O_3_ in the presence of Ag (Figure 5b), the binding energies of Ga 3d, O 1s, As 3d, and Ag 3d (with two peaks) are 20.8 eV, 532 eV, 47.6 eV, 368.4 eV and 371.7 eV, respectively. Both samples reveal peaks of Ga and O for Ga_2_O_3_ and Ag that are in agreement with the handbook of XPS spectra [37]. XPS analysis of the β-Ga_2_O_3_ on GaAs coated with Ag (Figure 5b) shows a positive shift due to the effect of absorbed oxygen [38].

The Ag3d peaks from metallic silver are known to be asymmetric, while the Ag3d peaks from Ag oxides are symmetric and display binding energies 0.8 eV below the metallic peak. The inlet for Ag3d binding energies in Figure 5b shows a metallic Ag peak at 368.8 eV and another broad band at about 371.2 eV. The difference in peak binding energies of 2.4 eV is larger than those of Ag oxides. Calderon et al. [39] have attributed the peak at 369.4 eV binding energy to Ag clusters smaller than 4 nm, which changes position and form depending on the sizes and distribution of the cluster [39,40,41]. Therefore, these results suggest that Ag catalyst forms both clusters and metallic bonds.

### 3.2. Growth Mechanism of β-Ga_2_O_3_ NWs on GaAs in the Presence of Ag

**Figure 6:**
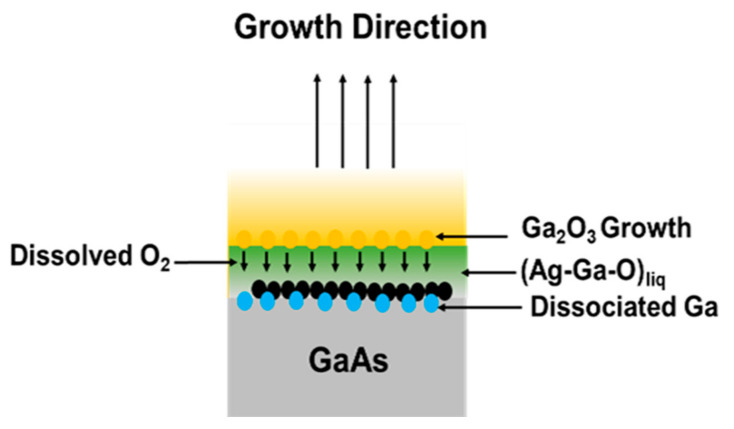
The growth mechanism of Ga_2_O_3_ NWs on GaAs substrate in the presence of Ag. O_2_ in the Ag–Ga–O liquid phase at 1000 °C will be stripped by the dissociated Ga to enhance the growth of Ga_2_O_3_ NWs by VLS mechanism.

To understand the complex growth mechanism of very thin and long Ga_2_O_3_ nanowires by thermal oxidation of GaAs in the presence of Ag, we looked into the EDS mapping and the phase diagram of Ag–GaAs (phase diagram No. 1300896) [33]. The experimental results suggest that the oxidation of GaAs is a progressive process that includes simultaneous and sequential temperature-dependent processes. Figure 6 illustrates a possible growth mechanism for Ga_2_O_3_ nanowires on GaAs. Around 650 °C, the Ga–As phase diagram shows that GaAs decomposes into Ga_(l)_ and As_(g)_ [20,42], resulting in Ga_(l)_ droplets on the surface of the GaAs substrate. As is expected to evaporate at temperatures above 580 °C [43]. The phase diagram of Ag–GaAs at 800 °C shows that 6–14 at. % of GaAs becomes a liquid phase mixed with solid phase of Ag. At 1000 °C and above Ag melting temperature, Ag–Ga–As forms a liquid mixture.

Thermodynamics studies of Ag–O and Ga–O systems show the solubility of oxygen in solid Ag [44] is much higher than that in liquid gallium [45]. Residual oxygen molecules in the chamber continue to react with Ag. Concomitantly, more oxygen diffuses into Ag. At higher temperatures above 626 °C, annealing Ag catalyst leads to a high concentration of O_2_ in Ag [46]. Originally, O_2_ atoms are adsorbed in Ag catalyst and then desorbed or diffused by volume diffusion. Ag thin film serves as a catalyst during oxidation by absorbing more oxygen than Ga would be able to absorb by itself [21]. At all temperatures, the oxidation enthalpy of Ga is much higher than that of Ag. Thus, it is expected that Ga nucleation begins to occur on Ag. As the diffusion length of Ga increases with temperature, more Ga atoms diffuse to Ag [25]. Hence, liquid Ga extracts oxygen from the Ag reservoir where more oxygen is available in the interface to react with Ga. Therefore, the formation of Ga_2_O_3_ crystals will be enhanced at the interface of the Ag–Ga–O liquid mixture and Ga_2_O_3_, leading to an equilibrium phase of AgGaO_x_. Consequently, Ga_2_O_3_ begins to nucleate with high density at 800 °C. However, at 1000 °C, Ga_2_O_3_ nanowires continue to grow as the diffusivity and solubility of oxygen molecules increase with temperature, leading to denser and longer nanowires (Figure 2) due to supersaturation of the species.

Generally, the nucleation mechanism of the oxide nanowires and the mass transport mechanism govern the nanowire oxide growth [47]. The oxidation of GaAs at high temperature results in a film that detaches from the GaAs surface due to the lattice mismatch and the large compressive thermal expansion generated at the Ga_2_O_3_/GaAs interface during heating. Since Ga_2_O_3_ and GaAs differ in molar volume and structure, the change in the local volume generates stress at the Ga_2_O_3_/GaAs interface which would serve as a driving force for outward diffusion of Ga atoms. Additionally, the tensile stress generated in the growing film enhances nanowire nucleation by reducing the lattice mismatch strain at the interface [48]. During the nanowire oxide growth, the sharp tip of the nanowires suggests a diffusion from bottom to tip, terminating as the liquid mixture of Ag–Ga–O is condensed into the solid state Ga_2_O_3_ by cooling path [49].

**Figure 7:**
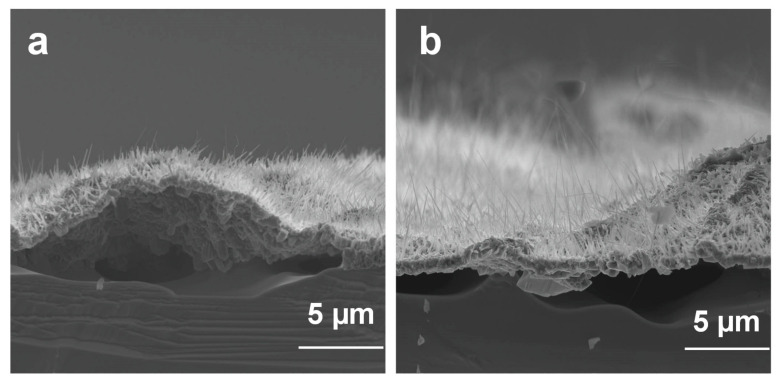
Side views of the delaminated film of Ga_2_O_3_ nanowires grown on GaAs at 1000 °C. (**a**) Ga_2_O_3_ nanowires grown on N-type GaAs substrate with (111) orientation and (**b**) P111. Film of Ga_2_O_3_ delaminated due to the different thermal expansion coefficient and lattice mismatch.

Different cracks at the surface of the grown Ga_2_O_3_ film of nanowires were observed due to the difference in lattice mismatch and the coefficients of thermal expansion between GaAs and Ga_2_O_3_ that causes large compressive stresses on the film as it grows [19]. During cooling after the thermal oxidation, the oxide film is subjected to particularly high compressive strains because of the large difference in thermal expansion coefficient between GaAs and β-Ga_2_O_3_ [19]. The induced stress provides the required energy to enhance the film delamination. Film stress is one of the main issues that causes defects and failure in the thin film technology. As the growth temperature increases, the large difference in coefficients of thermal expansion becomes more significant. Therefore, the film may be subjected to extreme stress, resulting in delimitation and cracking. Significant delamination was also visible in the side view of the SEM images in Figure 7.

#### STEM

**Figure 8:**
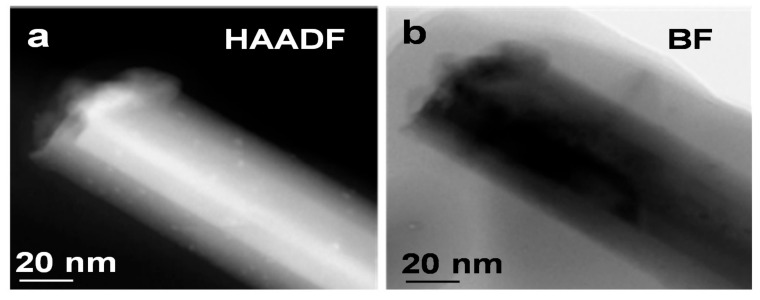
(**a**) High-angle annular dark-field and (**b**) bright field (BF) STEM images of a Ga_2_O_3_ NW grown on P(111) GaAs substrate at 1000 °C with Ag. Ga_2_O_3_ NW decorated with Ag NPs is observed in the images. This is attributed to surface diffusion of Ag thin film to Ga_2_O_3_ NW surface.

A 200 KeV JEOL JEM-2100AC scanning transmission electron microscope was further used to search for the silver that was detected in the XRD scans but not seen in the SEM/FIB investigation. STEM will be applied to define the internal crystal structure of the fabricated sample. The magnification can show the local structure of the samples with a resolution of 5 nm. Additionally, STEM provides better resolution, leading to a better observation and analysis of the sample’s internal structures and phases. STEM will assist in analyzing the crystallographic structure of the sample at an atomic scale. Figure 8 shows a STEM image of a Ga_2_O_3_ nanowire grown on the P111 sample coated with silver and oxidized at 1000 °C. The samples were prepared using a razor blade and collecting the nanowires on a TEM grid.

To explore the presence of Ag on the surface or at the tip of the grown Ga_2_O_3_ nanowires, STEM images of a Ga_2_O_3_ nanowire grown on the P(111) GaAs with Ag and oxidized at 1000 °C are shown in Figure 8. Ga_2_O_3_ nanowire in the presence of Ag contains a few nanoparticles that decorate the surface with diameters between 2 and 2.5 nm, causing some defects in the lattice. High-angle annular dark-field (HAADF) was used to evaluate the brightness of the nanoparticles. Due to the enhanced Z contrast, the nanoparticles are very bright and are expected to be Ag NPs.

The existence of Ag NPs that decorated the nanowires is expected due to the unconsumed Ag at the surface of Ga_2_O_3_ enabled by the thermal and surface diffusion of Ag film. This observation is consistent with the binding energies associated with the Ag clusters observed in the XPS of the β-Ga_2_O_3_ (Figure 5b). At higher temperature (1000 °C), gallium suboxide (Ga_2_O) could evaporate from the cracks which have been observed on the surface of Ga_2_O_3_. Subsequently, it could be reduced to Ga_2_O_3_ and liquid Ga. Ga could absorb the dissolved oxygen from Ag to form more Ga_2_O_3_ nanowires due to the high flux of oxygen at the interface of the Ag–Ga–O liquid mixture and Ga_2_O_3_ growth. Consequently, gallium suboxide (Ga_2_O) with the presence of Ag is expected to enhance the growth of denser, longer and thinner nanowires by the VLS mechanism.

### 3.3. Electrical Measurements of β-Ga_2_O_3_ NWs on N(111) Si-Doped and P(111) Zn-Doped GaAs

**Figure 9:**
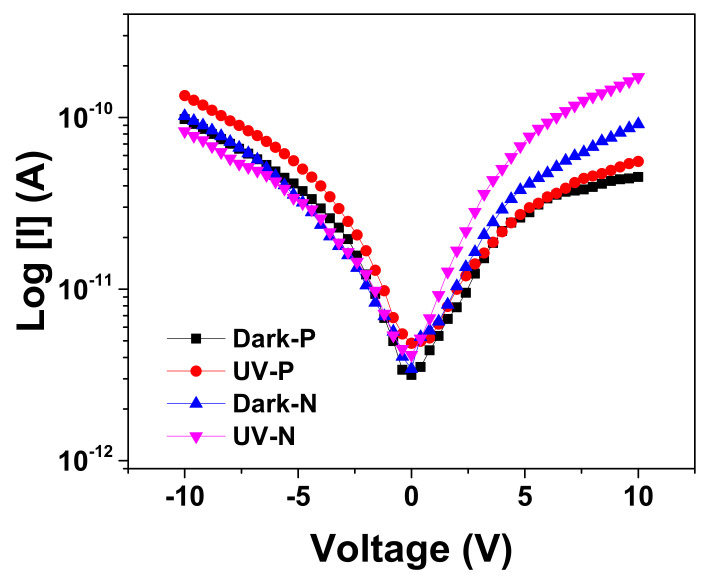
Semi-log scale of dark and photocurrent characteristics of Ga_2_O_3_ NWs grown on N(111) and P(111) GaAs at 1000 °C. Data were obtained as an average of the results obtained from 10 to 25 measurements on 3 samples.

Au/β-Ga_2_O_3_/Au metal-semiconductor-metal (MSM) photoconductor was fabricated to examine the electronic conductivity of the grown β-Ga_2_O_3_ nanowires. The current–voltage (*I–V*) characteristics (Figure 9) were measured at 10 V in dark conditions and under UV illumination for an MSM photodiode with β-Ga_2_O_3_ nanowires that were grown with Ag-coated P(111) Zn-doped GaAs and N(111) Si-doped GaAs, which were thermally oxidized at 1000 °C. It has been observed that the photocurrent and dark current of Ga_2_O_3_ nanowires grown at the surface of Si-doped GaAs were higher than that of nanowires grown on Zn-doped GaAs. In addition, no difference was obtained between the dark and photocurrent of Ga_2_O_3_ nanowires grown on Zn-doped GaAs.

Certainly, the assessment of the background conductivities is difficult to evaluate unless several different techniques are performed. Generally, the introduction of either n- or p-type dopants can determine if the current is carried by electrons or holes. However, more research needs to be carried out to explore the effects of impurities and minimize them. Silicon is one of the major impurities. It is found to strongly correlate to n-type conductivity [50]. If the silicon is incorporated during oxidation, it could increase the n-type conductivity of the film. Generally, the p-type behavior in β-Ga_2_O_3_ is still a challenge. Based on the element group, Zn could be a deep acceptor and will compensate oxygen donor states [51].

Even though the oxidation method of GaAs is simple and inexpensive to produce β-Ga_2_O_3_ nanowires, it does not allow precise control of nanowire growth, hence their lack of directionality. If this nanowire-based device is used as a sensor, this means that the effective sensing surface area is reduced. In addition, the challenge of how to re-use the grown Ga_2_O_3_ and transfer it onto a different substrate to fabricate a device with a minimum deformation and high-quality coating needs to be addressed.

## 4. Conclusions

In this study, two different GaAs samples (n-type Si-doped mono-crystalline GaAs (N111) and p-type Zn-doped monocrystalline GaAs (P111)) were characterized to study the oxidation of GaAs in the presence of Ag. At 1000 °C, denser, longer, and sharper nanowires were observed. XRD results showed that the [400] orientation becomes dominant during thermal oxidation and suggests that the nanowires might have preferential growth in the [400] direction. SEM images showed that dense and long nanowires were obtained with GaAs coated with silver and oxidized at 1000 °C. EDS detected a higher atomic percentage of oxygen in β-Ga_2_O_3_ nanowires grown on Ag-coated GaAs surfaces than on plain GaAs and also in samples exposed to higher temperatures, such as 1000 °C as opposed to 800 °C. We conclude that silver films promote the growth of Ga_2_O_3_ nanowires at temperatures around 1000 °C. High-angle annular dark-field (HAADF) by STEM revealed the presence of bright nanoparticles in the β-Ga_2_O_3_ nanowires exposed to the silver thin film catalyst. These are likely to be silver nanoparticles due to the self-diffusion effects. Silver has a great impact on increasing oxygen absorption and facilitating dense and long nanowire growth because of its high diffusivity and solubility in oxygen at higher temperatures. However, deformation and delamination were observed on the surface of the Ga_2_O_3_ nanowire film, which could be the result of various factors such as the thermal expansion coefficients, film stress and lattice mismatch. For UV detection, the sample of n-type Si-doped mono-crystalline GaAs (N111) has shown better conductivity compared to p-type Zn-doped monocrystalline GaAs (P111). This research is important for the field of UV sensors, optoelectronics and high-power electronics. For sensing, Ga_2_O_3_ nanowire grown using Ag catalyst has great potential for power electronics applications with its high sensitivity and fast response time. In particular, resistive gas sensing applications at high temperatures have been studied intensely [52,53,54,55]. Gas sensors that can operate at high temperatures are essential for monitoring the concentration of exhaust gases that could cause harm upon exposure or lead to explosions and fires.

## Figures and Tables

**Table 1:** Measured (this work) and reported average diameter, length and morphology of nanowires based on different catalysts, techniques and substrates.

Catalyst		Technique	Substrate	Avg. Nanowire	
				Diameter (nm)	Length
**Experimental Results obtained in this work**					
No Catalyst		Oxidation	GaAs	250–400 *	800 nm–1.35 µm *
Ag Thin Film				25–40 *	Several tens of µm *
**NPs**	**Ref.**	**Reported Results**			
Free Catalyst	[12]	CVD	Ga/Ga_2_O_3_ mixture and O_2_	40–70	10–20 µm
	[28]		Si/SiO_2_ and Al_2_O_3_	50–75	>10 µm
Ag NPs	[21]	Sol–gel and VLS	GaAs	18–30	Several tens of nm to a few hundred µms
Au NPs	[29]	Thermal VLS	Au-pretreated Si	34	20–60 nm
Ni NPs	[28]	CVD	Ga/Ga_2_O_3_ mixture and O_2_	30–80 with an average value of 50	up to 500 µm
Pt NPs	[30]	Thermal decomposition, oxidation and epitaxy	GaN films on sapphire	∼80	Several tens of µm

* Data were obtained as an average of the results obtained from 10 to 25 measurements on 3 samples.

**Table 2:** Elemental composition of Ga_2_O_3_ NWs on GaAs substrates coated with and without Ag and oxidized at 800 °C and 1000 °C. The results suggest that the atomic percentages of Ga and O increase as temperature increases. However, the atomic percentages of As and Ag get reduced with increased temperature.

Composition (+/−)	800 °C				1000 °C			
	O	Ga	As	Ag	O	Ga	As	Ag
N(111)-No Ag	37.3	31.9	30.8	0.0	54.7	45.3	0.1	0.0
N(111)-Ag	47.1	32.4	12.9	7.6	54.8	45.2	0.0	0.0
P(111)-No Ag	38	31.0	29.8	1.2	54.8	45.1	0.1	0.0
P(111)-Ag	42.5	29.3	18.5	9.8	54.8	44.9	0.3	0.0
